# Functional analysis of epilepsy-linked pathogenic variants of the Munc18-1 gene in the inhibitory nervous system of *Caenorhabditis elegans*

**DOI:** 10.17912/micropub.biology.001174

**Published:** 2024-04-22

**Authors:** Keiko Gengyo-Ando, Akane Osawa-Noguchi, Hideki Ando, Junichi Nakai

**Affiliations:** 1 Oral Physiology, Tohoku University Graduate School of Dentistry, Miyagi, Japan; 2 Brain Science Institute, Saitama University, Saitama, Saitama, Japan

## Abstract

Heterozygous de novo mutations in Munc18-1, which is essential for neurotransmitter release, cause early infantile epileptic encephalopathy. Munc18-1-linked epilepsy is currently an untreatable disorder and its precise disease mechanism remains elusive. Here, we investigated how Munc18-1 pathogenic variants affect inhibitory neurons using *Caenorhabditis elegans* . Expression analysis revealed that three missense mutant proteins form aggregates in the cell body of gamma-aminobutyric-acid (GABA)-ergic motoneurons, resulting in a strong reduction of their expression in axons. Their defects of axonal expression correlated closely with pentylenetetrazol-induced convulsions, suggesting that the degree of instability of each mutant protein account for the severity of the epileptic phenotypes.

**Figure 1. Effects of unc-18 mutations orthologous to pathogenic Munc18-1 mutations on expression of UNC-18 protein and GABAergic function in C. elegans:**
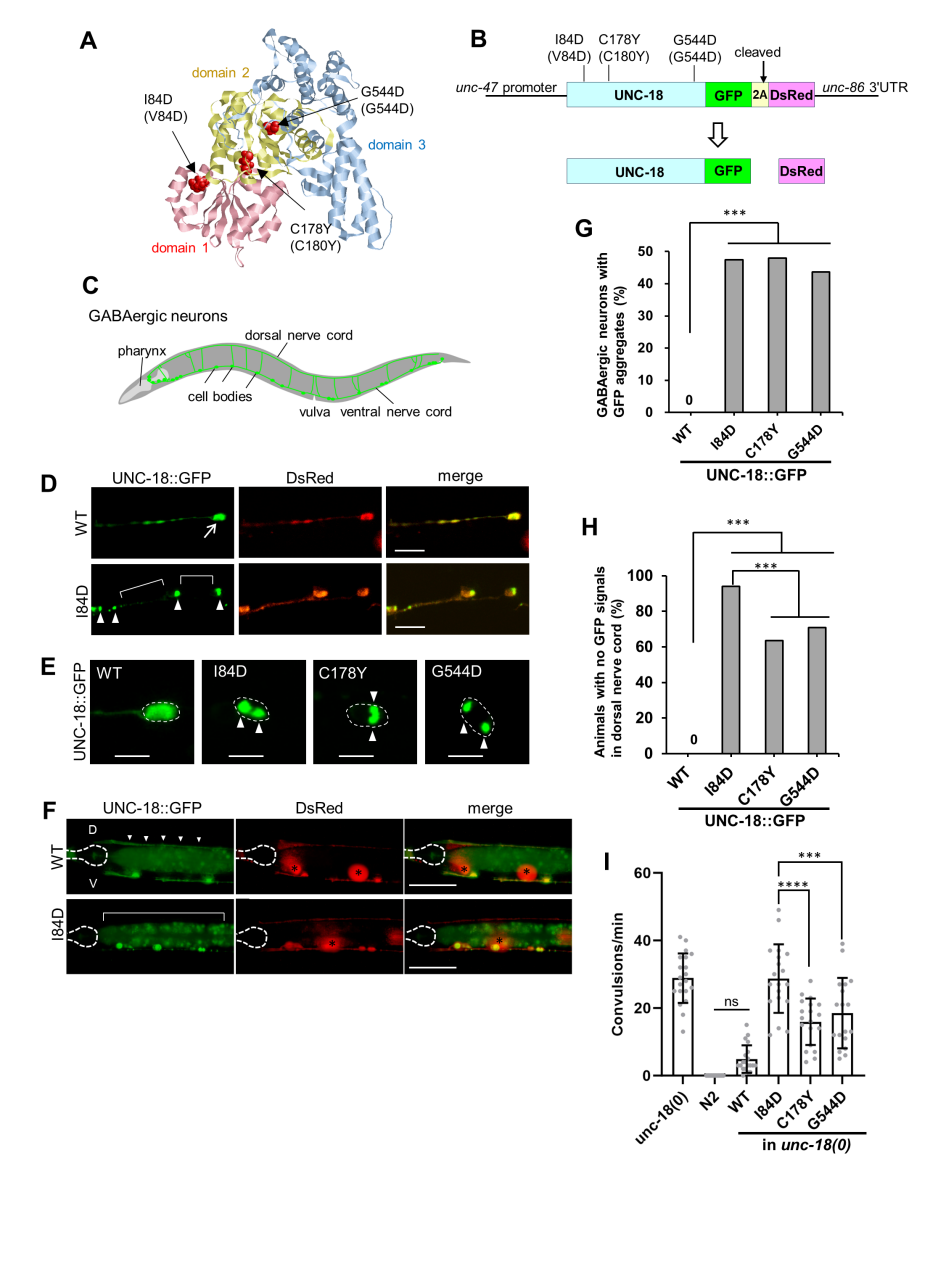
(A) Three UNC-18 missense mutations analogous to pathogenic Munc18-1 mutations. Each mutated residue is shown in a space-filling representation in a 3D model of UNC-18. The Sec1/Munc18 family protein consists of three domains (domain 1: pink, domain 2: yellow, domain 3: blue). I84D is located in domain 1 and C178Y and G544D are located in domain 2. (B) Schematic diagram of a DNA construct for a 2A peptide (T2A)-mediated co-expression system. In this system, UNC-18::GFP and DsRed are theoretically expressed in a 1:1 molar ratio in GABAergic neurons. (C) Schematic representation of the GABAergic nervous system of *C. elegans* . (D) Expression patterns of the transgenic lines harboring the UNC-18 (WT)::GFP::T2A::DsRed (upper panels, *jqEx248* ) and the UNC-18 (I84D)::GFP::T2A::DsRed (lower panels, *jqEx252* ). WT proteins showed a soluble distribution pattern throughout the axons and the cell bodies (arrow) in the ventral nerve cord. In the mutant transgenic strain, DsRed was distributed normally in the ventral nerve cord and cell bodies, whereas I84D protein exhibited a punctate pattern (arrowheads) and a reduced expression in axons (brackets). Scale bars, 10 µm. (E) Aggregation of mutant UNC-18 proteins in the cell bodies of GABAergic neurons. In the transgenic lines expressing GFP-tagged mutant UNC-18, large aggregates (arrowheads) were observed. WT: *jqEx248* , I84D: *jqEx252* , C178Y: *jqEx262* , G544D: *jqEx258* . Scale bars, 5 µm. (F) Reduced expression of mutant UNC-18 proteins in the axons. WT proteins are distributed throughout the dorsal nerve cord (arrowheads), whereas mutant proteins are not detected (bracket). Dashed lines indicate the boundaries of the pharynx. Note that the signals in the intestine in the GFP channel are from the autofluorescence of the intestinal granules (Hermann et al., 2005). D: dorsal, V: ventral. Asterisks indicate coelomocyte RFP co-injection marker. Scale bars, 50 µm. (G) Quantification of percentage of GABAergic cell bodies with aggregates (n=98-110 from 11-13 animals). ***p<0.001. Statistics: Fisher's exact test, two-tailed. (H) Quantification of percentage of animals with no GFP signals in the dorsal nerve cord (n=101-113). ***p<0.001. Statistics: Fisher's exact test, two-tailed. (I) PTZ assay. The number of convulsion per minute were measured in *unc-18(e81)* , N2, WT: *unc-18(e81);jqIs21* , I84D: *unc-18(e81);jqIs23* , C178Y: *unc-18(e81);jqIs9* , and G544D: *unc-18(e81);jqIs43* . A higher PTZ index (the number of spasms per minute) represents more severe GABAergic defects. mean±SD. Statistics: one-way ANOVA followed by Tukey's multiple comparisons test. ***p<0.001.****p<0.0001. ns: not significant.

## Description

Early infantile epileptic encephalopathy (EIEE) with suppression burst, also known as Ohtahara syndrome, is the earliest form of age-dependent epileptic encephalopathy (Yamatogi and Ohtahara, 2002) . Recent high-throughput sequencing studies have identified a number of heterozygous de novo mutations in the Munc18-1 gene in patients with EIEE type 4 (EIEE4), a severe form of epilepsy associated with developmental delay and intellectual disability (Saitsu et al., 2008; Deprez et al *.* , 2010; Pavone et al., 2012). Currently, seizures in EIEE4 are largely resistant to antiepileptic drugs and there is no targeted therapy for EIEE4. Munc18-1 (also known as STXBP1) encodes a neuronal Sec1/Munc18 protein that regulates synaptic vesicle exocytosis in both vertebrates and invertebrates (Gengyo-Ando et al., 1993; Hata, Slaughter and Südhof, 1993; Harrison et al., 1994). Imbalance between the inhibitory tone and neural excitation, especially perturbation of the inhibitory tone, is strongly suspected as the cause of epilepsy, but how the epilepsy-linked Munc18-1 variants affect inhibitory neurons *in vivo* is poorly understood. Here, we investigated the effects of pathogenic Munc18-1 missense mutations on protein expression and GABAergic neurotransmission in the *C. elegans* inhibitory nervous system.

*C. elegans* UNC-18 (Munc18-1 homologue) was originally isolated by paralyzed phenotype (Brenner, 1974) and is essential for neurotransmitter release including acetylcholine and GABA (Hosono et al., 1992) . We investigated here the three pathogenic Munc18-1 mutations, V84D, C180Y and G544D (I84D, C178Y, G544D for UNC-18 ; Figure 1A ), which were first identified in 2008 in patients with Ohtahara syndrome (Saitsu et al., 2008) . To evaluate the expression of these mutant proteins, we used a 2A peptide-mediated co-expression system, which allows the expression of multiple proteins within a single open reading frame through a co-translational cleavage event (Luke et al *.* , 2008; Ahier and Jarriault, 2014). We generated transgenic lines expressing GFP-tagged wild-type (WT) or mutant UNC-18 analogous to the pathogenic Munc18-1 mutant with DsRed under the *unc-47* (a vesicular GABA transporter) promoter ( Figure 1B ). We chose the *unc-47* promoter so that transgene products would be specifically expressed in GABAergic neurons, i.e., the inhibitory nervous system of *C. elegans* ( Figure 1C ). We injected these GFP-tagged UNC-18 constructs following our routine protocol in which coelomocyte RFP maker was co-injected. GFP-tagged UNC-18 (WT) showed a soluble distribution pattern throughout the GABAergic motoneurons in the ventral/dorsal nerve cords and cell bodies, similar to soluble DsRed ( Figure 1D upper panels ). On the other hand, GFP-tagged mutant UNC-18 (I84D) exhibited a punctate pattern that differed from the soluble distribution ( Figure 1D lower panels ). This observation is consistent with previous reports showing the aggregation of Munc18-1 mutant proteins in cultured cells and animal models (Chai et al., 2016; Guiberson et al., 2018) . We found that in all three missense mutants, approximately 50% of GABAergic cell bodies in the ventral nerve cord contain aggregates of mutant proteins (n=98-110 from 11-13 animals, Figure 1E and G ). Furthermore, a strong reduction of their expression in the axons was observed in all transgenic strains expressing the mutant proteins ( Figure 1F ). In the dorsal nerve cord, I84D (V84D in Munc18-1) showed the most reduced expression, with no GFP signal detectable in 94.1% of animals ( Figure 1H ).

In order to examine the functional defects of the pathogenic mutants, we generated transgenic lines introducing wild-type (WT) or mutant (I84D, C178Y and G544D) *unc-18* gene in an *unc-18 ( e81 )* null mutant background (hereafter referred to as *unc-18 (0)* ) and performed pentylenetetrazole (PTZ)-induced seizure assay. PTZ is a GABA _A_ receptor antagonist and is commonly used to assess defects in GABAergic signaling in animal models, including *C. elegans* (Bessa, Maciel and Rodrigues, 2013) . PTZ treatment induced rapid head seizures in *unc-18* null mutant animals ( Figure 1I ), while it had no seizure-inducing effect in wild-type N2 animals as described (Williams et al., 2004) . The increased sensitivity to PTZ observed in *unc-18* *(0)* was completely rescued by *unc-18* (WT) expression; WT controls ( *unc-18 (0); unc-18 (WT)* ) showed slight convulsions, but not significantly different from wild-type N2 (p=0.29). In contrast, all three mutants showed significantly higher sensitivity to PTZ than the WT control (WT, 4.9±4.1, n=20; I84D, 30.5±12.6, n=20; C178Y, 15.9±6.8, n=19; G544D, 18.5±10.4, n=18; I84D and G544D to WT, p<0.0001; C178Y to WT, p<0.001, Figure 1I ), suggesting that the pathogenic mutants have reduced rescue activity for presynaptic GABA release defects compared to WT. Notably, as in the expression analysis above ( Figure 1H ), I84D (V84D in Munc18-1) again had the most severe effect as compared to the other mutants (I84D to C178Y, p<0.0001; I84D to G544D, p<0.001).

In this study, we showed that the disease-associated Munc18-1 mutant proteins formed aggregates in the cell body of GABAergic motoneurons and that their expression was severely reduced in axons. This result is consistent with the previous findings that the haploinsufficiency of Munc18-1 is one of the major causes for EIEE4 (Saitsu et al., 2010) . Importantly, we observed a close correlation between defects of axonal expression of mutant proteins and the GABA synaptic transmission phenotype. I84D (V84D in Munc18-1) was found to be the most severe mutation in both aspects, which is consistent with previous reports that V84D cause strong phenotypes in both mice and a humanized *C. elegans* model (Kovačević et al., 2018; Zhu et al., 2020). Recently, Munc18-1 haploinsufficiency has also been shown in organotypic slice cultures of the human subplate region; reduction of Munc18-1 by shRNA interference resulted in a reduction of glutamatergic synapse, with a compensatory increase in GABAergic synapse (McLeod et al, 2022) . Thus, Munc18-1 could have roles regulating the number of at least the two types of synapses and, in any case, requires a certain level of expression to exert its proper function. The difference in severity between Munc18-1 mutations may be caused by the degree of protein instability and/or the ability of WT and mutant Munc18-1 to co-aggregate. Recent studies suggest that EIEE4 is caused by a dominant-negative effect of mutant Munc18-1, i.e. by co-aggregation of mutant Munc18-1 with wild-type Munc18-1 or other synaptic proteins (Chai et al., 2016; Guiberson et al., 2018; Lanoue et al., 2019) . The system developed here will be used to further address this question.

## Methods

***C. elegans* strains and maintenance:** *C. elegans* strains were cultured on Nematode Growth Medium (NGM) seeded with *E. coli* OP50-1 as described (Brenner, 1974) . The wild-type strains Bristol N2 and *unc-18 ( e81 )* were obtained from the Caenorhabditis Genetics Center. The transgenic strains generated in this study are shown in Reagents.

**Molecular cloning:** *unc-18* genomic constructs containing *unc-18* mutations orthologous to pathogenic Munc18-1 missense mutations were generated as follows. Briefly, amino acid substitutions I84D, C178Y and G544D (V84D, C180Y and G544D in Munc18-1) were introduced into the *unc-18* genomic clone PE10 (Hosono et al., 1992) by mutating the respective codons using the QuickChange Lightning Site-Directed Mutagenesis Kit (Agilent). For the 2A peptide co-expression system, the *unc-18 (WT)::GFP::T2A::DsRed* fusion cDNA was generated by sewing PCR and cloned into the pFX_EGFPT vector (Gengyo-Ando et al., 2006) with the *unc-47* promoter (1.2 kb). Enhanced GFP (EGFP) was used for the GFP. I84D, C178Y and G544D were introduced into *unc-18 (WT)* cDNA as described above. All constructs were verified by DNA sequencing.

**Generation of transgenic strains:** DNA construct (20 ng/µl) and red fluorescent protein (RFP) markers (50 ng/µl) were co-injected into the gonads of N2 hermaphrodites using a standard protocol (Mello et al., 1991) . Stable transgenic lines carrying extrachromosomal arrays were obtained at the F2 generation and maintained. Integration of extrachromosomal arrays was performed by a UV irradiation method (Mitani, 1995) . We used transgenic lines with almost equivalent DsRed intensity between strains in our experiments. Integrated lines were backcrossed four times to N2 and then replaced by crossing into the *unc-18 ( e81 )* null mutant background. The transgenic strains generated in this study are shown in the reagents.

**Expression analysis:** We examined aggregates of UNC-18::GFP proteins in the cell bodies where DsRed was expressed. For each strain, 98-110 GABAergic cell bodies in the ventral nerve cord from 10-13 animals were examined and the percentage of cell bodies with aggregates was calculated. We observed UNC-18::GFP in the dorsal nerve cord anterior to the vulva where DsRed was expressed. For each strain, 101-113 animals were examined and the percentage of animals with no detectable GFP signal was calculated.

**Imaging:** L4 animals were mounted on 2% agarose pads and immobilized with 50 mM sodium azide. A Nikon confocal microscope (A1R, Nikon) with a 20x objective (Plan Apo Lambda, Nikon) and 5x digital zoom or an Olympus microscope (BX50, Olympus) with a 100x objective (UPlan Apo, Olympus) was used to image aggregates in the cell bodies. UNC-18 mutant aggregates were identified as GFP aggregates with a minimum diameter size of 0.5 µm. Images were captured with a Nikon digital camera (DS-Qi1Mc, Nikon). For imaging dorsal nerve cord, a Nikon microscope (ECRIPSE Ni, Nikon) with a 40x objective (Plan Apo Lambda, Nikon) was used. Images were captured with a DP28 digital camera (Olympus).

**Convulsion assay:** The PTZ assay was performed basically as described (Williams et al., 2004) . NGM plates containing PTZ (Sigma, P6500) at a final concentration of 5 mg/mL were freshly prepared and seeded with OP50-1 . Synchronized young adult animals were placed in the PTZ assay plate for 1 minute. After 1 minute of exposure, head-bobbing convulsions were recorded over 1 minute at 15 frames/sec using an Olympus DP21 digital camera attached to a stereomicroscope (SZX16, Olympus). The convulsions were counted and the number of convulsions per minute was measured. Approximately twenty animals of each strain were observed and analyzed.

**3D model** : Prediction of the 3D structure of *C. elegans* UNC-18 was performed using the I-TASSER server (https://zhanggroup.org/I-TASSER/) (Zhang, 2008) . The domain structure of C. elegans UNC-18 was based on the three-domain structure of Sec1/Munc18 proteins defined by Misura et al. (Misura, Scheller and Weis, 2000) . The structure was visualized with RasMol (version 2.7.5.2) ( http://www.openrasmol.org/ ).

**Statistics** : Differences in percentage of cell bodies with aggregates and differences in percentage of animals with no GFP signals were analyzed using Fisher's exact test in Excel Tokei (Social Survey Research Information) ( Fig. 1G and H ). Statistical significance in the number of convulsions was analyzed using one-way ANOVA followed by Tukey's multiple comparison test in GraphPad Prism 10.0.3 (GraphPad Software) ( Fig. 1I ).

## Reagents

**Table:** 

Strain	Genotype	Source
QJ3012	*unc-18 ( e81 )X; jqIs21 [ unc-18 (WT)+unc-122p::mCherry] *	This study
QJ3014	*unc-18 ( e81 )X; jqIs23 [ unc-18 (I84D)+unc-122p::mCherry] *	This study
QJ3011	*unc-18 ( e81 )X; jqIs9 [ unc-18 (C178Y)+myo-3p::DsRedm] *	This study
QJ3021	*unc-18 ( e81 )X; jqIs43 [ unc-18 (G544D)+unc-122p::mCherry] *	This study
QJ1221	*jqEx248 [ unc-47 p:: unc-18 (WT)::GFP::T2A::DsRed +unc-122p::mCherry] *	This study
QJ1225	*jqEx252 [ unc-47 p:: unc-18 (I84D)::GFP::T2A::DsRed +unc-122p::mCherry] *	This study
QJ1235	*jqEx262 [ unc-47 p:: unc-18 (C178Y)::GFP::T2A::DsRed +unc-122p::mCherry] *	This study
QJ1231	*jqEx258 [ unc-47 p:: unc-18 (G544D)::GFP::T2A::DsRed +unc-122p::mCherry] *	This study
